# Non-breeding changes in at-sea distribution and abundance of the threatened marbled murrelet (*Brachyramphus marmoratus*) in a portion of its range exhibiting long-term breeding season declines

**DOI:** 10.1371/journal.pone.0267165

**Published:** 2022-04-21

**Authors:** Scott F. Pearson, Ilai Keren, Monique M. Lance, Martin G. Raphael

**Affiliations:** 1 Science Division, Washington Department of Fish and Wildlife, Olympia, Washington, United States of America; 2 US Forest Service Pacific Northwest Research Station, Olympia, Washington, United States of America; MARE – Marine and Environmental Sciences Centre, PORTUGAL

## Abstract

The marbled murrelet (*Brachyramphus marmoratus*) is classified as a threatened species under the US Endangered Species Act in Washington, Oregon, and California USA due to population declines, loss of breeding habitat, and other factors. To date, population assessments have focused on breeding season at-sea surveys. Consequently, there is little information on this species’ distribution, abundance, and population trends during the non-breeding season, when murrelets are found exclusively in the marine environment. To address this information need, we assessed non-breeding (Sep—Mar) at-sea murrelet abundance patterns and population trends over 8 years, in a portion of its range where breeding season surveys indicate a 20-year population decline, Puget Sound, Washington, USA. This allowed us to assess whether non-breeding population trends mirrored those observed during the breeding season suggesting regional year-round conservation concerns and to also identify important over-wintering areas (areas of high abundance). We integrated our non-breeding abundance information with breeding season information to assess year-round patterns of abundance. This allowed us to test the prediction that murrelets move into the relatively protected inner marine waters of Puget Sound from harsher outer coastal habitats during the non-breeding season to molt and over-winter. Similar to trends from the breeding season, we observed strong murrelet density declines across the entire non-breeding period (Sep and Apr) with declines most pronounced in the fall and early winter (lateSep–Dec) survey windows when birds molt and in the spring just prior to breeding (Mar-Apr). Despite these declines, there was essentially no change in murrelet density in mid-winter (January—February) when overall density was lower. Puget Sound murrelet density exhibited a strong north-south gradient with relatively high densities to the north and low densities to the south; murrelets were largely absent from Central Puget Sound. For strata other than Central Puget Sound, density varied seasonally with birds more evenly distributed among strata between September and December but in the late winter/early spring period (Jan–Apr), murrelets were largely absent from all strata except the most northerly Admiralty Inlet Stratum, which appears to be important to murrelets year-round. Depending on the year, non-breeding season densities were nearly the same or higher than breeding season densities indicate that murrelets were not moving into the relatively protected inner marine waters of Puget Sound from more outer coastal environments during the non-breeding season as predicted.

## Introduction

Seventy percent of the world’s seabird populations monitored between 1950 and 2010 are declining [1]. The marbled murrelet (*B*. *marmoratus*), a seabird in the family Alcidae, is a federally threatened species [2] that exhibited range-wide declines south of the Washington–British Columbia border between 2000 and 2010 [3]. However, since 2010 populations have increased in portions of its breeding range in Oregon and northern California but continue to decline in the US portion of the Salish Sea (Puget Sound, Strait of Juan de Fuca and Strait of Georgia) [4].

Seabirds breed on land and yet, they spend most of their lives and obtain their food from the marine environment. As a result, their distribution and abundance at-sea during the breeding season is influenced by both terrestrial and marine factors [5–7]. Marbled murrelet at-sea distribution during the breeding season tends to be nearshore, in relatively cool waters with less human activities, and in proximity of larger areas of cohesive older forest nesting habitat [7, 8]. However, we know relatively little about the distribution and abundance of this species during the non-breeding season when it is exclusively tied to the marine environment. Overall, the conservation of marbled murrelets may hinge on protecting not only nesting habitat, which has been the focus of conservation efforts to date, but also on their marine habitat where they spend most of their lives.

Unlike most previous work, our research focuses on the non-breeding season when murrelets are not necessarily central place foragers and it also focuses on a time of year when we have little to no information on distribution and abundance and when they spend all their time at-sea. We also focus our work on the portion of the murrelet’s listed range, Puget Sound USA, where it is exhibiting long-term and consistent declines [4]. This new research allowed us to determine if breeding season trends are reflective of non-breeding season trends and to identify important over-wintering areas. Specifically, we assessed late September to early April abundance patterns in four “seasons” (late-Sep—Oct, Nov—Dec, Jan—Feb, and Mar—early-Apr) and four geographic strata across 8 years. We also integrated our non-breeding abundance information with breeding season data to assess whether murrelets are moving into the relatively protected inner marine waters of Puget Sound from harsher outer coastal habitats during the non-breeding season to molt and over-winter. In other words, is Puget Sound an important region for over-wintering and molting birds? The limited research on non-breeding movements indicates that murrelets continue to primarily use nearshore environments with some staying in their breeding region year-round, while others appear to disperse (both to the north and to the south depending on the geographic area assessed) from breeding locations to molt and over-winter elsewhere [9–12].

## Materials and methods

### Study area and species

The Salish Sea is a 16,925-km^2^ inland sea extending from Olympia, Washington, USA, north to Campbell River, British Columbia, Canada, and includes Puget Sound, the Strait of Georgia, and the Strait of Juan de Fuca. It is bounded by mainland British Columbia and Washington State on the east, Vancouver Island and the Olympic Peninsula on the west, and includes 7,470 km of coastline. This research occurred primarily in the Puget Sound portion of the Salish Sea.

Puget Sound has been described as a fjord-estuary complex with multiple deep-water basins (depths in our study area reach > 240 m) separated from each other by shallower sills. The interaction of freshwater inputs (thousands of rivers and streams), tidally driven currents and the unique bathymetry of the region ultimately influences patterns of upwelling and downwelling, freshwater mixing, and dissolved oxygen and salinity levels [13] which, in-turn, influence productivity and species composition and structure. This region is biotically rich [14–16] but like many highly urbanized estuaries and coastal areas, Puget Sound faces many human-caused threats including contaminants [17, 18], increased human activity and marine traffic [19], as well as shoreline hardening, and loss of estuarine habitat through diking [20]. These human impacts have the potential to influence murrelet food resources, daily activity patterns and ultimately survival [21].

Murrelets are pursuit-divers that forage on small pelagic schooling fish and large zooplankton. Throughout their listed range, they primarily nest in coastal, old-growth coniferous forests. Overall, the conservation of marbled murrelets may hinge on protecting not only nesting habitat, which has been the focus of conservation efforts to date, but also on their marine habitat where they spend most of their lives. To better understand important marine habitats for marbled murrelets, we focus on the non-breeding distribution and abundance at-sea when they are not necessarily central place foragers and during a time of year when we have little to no information on distribution and abundance and spend all their time at-sea.

### Field methods

**Fig 1:**
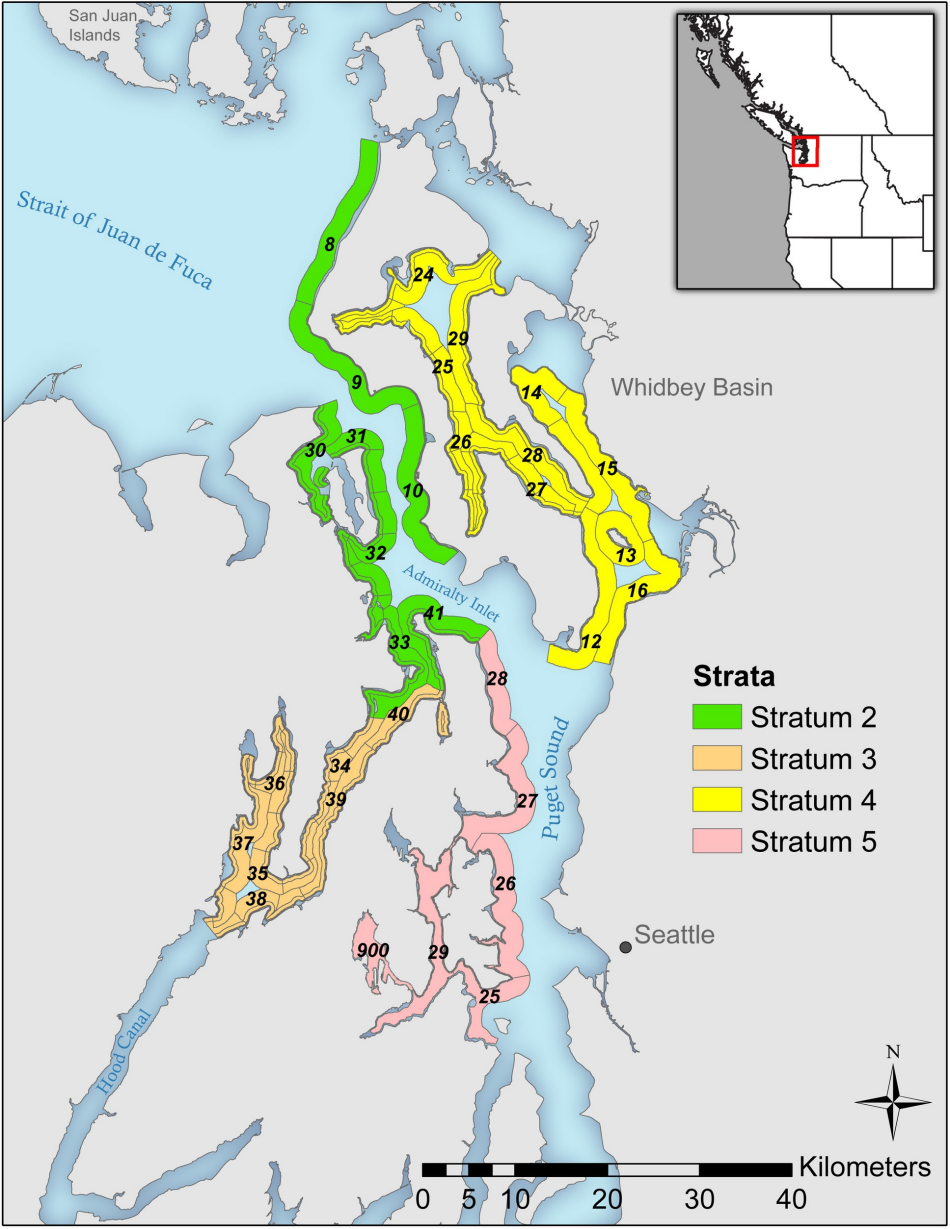
Survey area and design. Stratum and primary sampling unit (PSU) locations in Puget Sound. Strata are defined in the legend and PSUs are numbered on the map.

We conducted line transect [22] non-breeding surveys, generally following the methods of [23] between the fall of 2012 and the spring of 2020. Breeding surveys were conducted as part of the Northwest Forest Plan Effectiveness Monitoring Program between (2012–2016, 2018). The non-breeding sampling frame consisted of four strata in central to northern Puget Sound (Fig 1). We used the same Primary Sampling Units (PSUs) developed by [23] but stratified the PSUs differently. We stratified the sampling area based on murrelet density (see [23]), oceanographic basins in the US portion of the Salish Sea (https://www.eopugetsound.org/articles/geographic-boundaries-puget-sound-and-salish-sea). Our strata roughly correspond with the Salish Sea basins as follows: Stratum 2 is Admiralty Inlet (n = 8 PSUs), Stratum 3 is North Hood Canal (n = 7 PSUs), stratum 4 is the Whidbey Basin (n = 11 PSUs), and Stratum 5 is Central Puget Sound (n = 6 PSUs). Our survey design consisted of surveying each PSU in the following time intervals or “seasons”: late-Sep—Oct, Nov—Dec, Jan—Feb, and Mar—early-Apr. To avoid spatial and temporal clustering, we randomly select Stratum x PSU × day combinations to survey within each of these approximate 2-month “seasons” and alternated the direction of travel for each PSU sampled.

The shape, design (including justification for the design), location and sampling strategy for our PSUs was identical to that of [23] and we used a subset of the PSUs from [23] to allow direct comparison with breeding season surveys. For the Northwest Forest Plan Effectiveness Monitoring Program, [23] established a total of 98 PSUs that meet end-to-end along shore without any gaps for the entire US portion of the Salish Sea (Strait of Juan de Fuca, San Juan Archipelago, and Puget Sound). We used 32 of these PSUs for our research but defined our strata differently, as described above. Briefly, PSUs cover approximately 20 km of shoreline and are 2 km wide and were designed to sample the nearshore, where murrelets are generally more abundant, more intensively than the offshore where their density is lower (please see [23] for details).

The team of observers consisted of two observers, one data recorder, and a rotating boat operator on an 8-m long boat with raised observer platforms travelling at 8–12 knots (15–22 km/hr). Surveys were nearly always conducted in Beaufort 2 or less per the protocol [23]. If > 25% of the survey was in a Beaufort 3 or greater, the survey was not included in the analysis and the survey was repeated on a later date. Surveys were only conducted when surveyors can see a murrelet at least 150 m from the boat. During a survey, the two observers, one responsible for each side of the boat’s center line, reported observations (murrelet group size, distance) of all birds detected from 0° (strait ahead of the boat along the transect line) to 90° abeam to a data recorder in the cabin via wireless headsets. The data recorder entered the information directly into a laptop computer using DLOG2 software (developed by R.G. Ford, Inc., Portland, OR.) that is interfaced with a GPS unit that collected real-time location data for each observation. Transect survey length was calculated from the GPS trackline and was also recorded in DLOG2. Survey team training and distance testing follow our methods/protocol publication, [23] and are also described in [24]. No permits were required to conduct this research because no birds were handled or pursued, and surveys were conducted in US navigable waters. Our breeding season surveys are covered under WDFW’s Section 6 contract with US Fish and Wildlife Service.

### Analytical approach

Miller et al. [3] previously assessed the effects of observer and sea condition on density and trend estimates for this same geographic region. They found that no one model was consistently the best; each model (no-covariate, crew, sea condition, crew plus sea condition) performed best in at least one year. More importantly, they found almost no effect of covariate models on density or trend estimates (see [3]: Fig 5). This result is not surprising because sea-state effects are intentionally minimized in the survey protocol, which precludes surveys during rough seas (Beaufort force ≥3). In addition, a professional year-round survey team and regular distance testing resulted in greater consistency among years. Consequently, we did not examine covariate models here.

We used a Bayesian approach to estimating average murrelet density (murrelets per km^2^) with an associated estimate of precision for the target population in Puget Sound (strata 2–5). We constructed a hierarchical model in JAGS-4.2.0 [25] accessed through R-3.4.0 [26] to jointly estimate seasonal and annual trends in density, as well as seasonal distribution between strata.

Using this analytical approach, we developed two models. The primary model was our non-breeding model where we used all 32 PSUs distributed in four strata that were sampled once in four approximate 2-month intervals (late-Sept-Oct, Nov-Dec, Jan-Feb, Mar-early-Apr) for 8 non-breeding fall through spring years (See S1 Table). We used this model to estimate non-breeding season changes in murrelet group size, density and change in density across space (strata) and years. We also developed a year-round model using only the 11 PSUs and years (2014, 2015, 2017) when these PSUs were monitored in both the breeding and non-breeding season survey efforts. Because of the small sample of years (2014, 2015, 2017) when surveys occurred in both the breeding and non-breeding seasons, we focus our breeding—non-breeding comparison on changes in density and group size between breeding and non-breeding seasons. During the breeding season, all the 11 PSUs were sampled twice, once between 15 May and mid-June and a second time between mid-June and 31 July. To keep estimates consistent with approximate 2-month survey windows, we used May-June and June-July as our survey windows because the summer protocol specifies a survey of all PSUs during these two survey windows.

Bird counts in PSUs were modeled using a Poisson distribution where the estimated rate for each year, season, and stratum correspond to murrelet densities. We allowed the natural log of total density (weighted average of all strata) to vary by year with seasonal estimates within a year varying around a common annual mean. For the non-breeding season, seasonal natural log densities were further modeled as a function of stratum to estimate seasonal changes in spatial distribution of murrelets.

We accounted for imperfect detection of murrelet groups away from the transect line using a half-normal decreasing function of distance (in m). To improve the detection model, 4% of data was right-truncated prior to analysis by discarding observations made at distances greater than 210 m [22]. Gamma priors were placed on the shape parameters of half-normal detection functions in every season and year, and they were allowed to vary around a common mode for each year which, in turn, varied around a global mean.

Seasonal averages of group size were modeled with rescaled negative binomial priors to adjust for the absence of zero groups. For the most part, murrelets were detected as pairs or singles (67% and 25.5%, respectively, in the non-breeding season and 58% and 32.5%, respectively, in units sampled year-round). Therefore, we did not attempt to model the effect of group size on detection by distance, instead we used the estimated average group sizes to adjust estimated group encounter rates back to the number of individuals.

We placed diffused (non-informative) priors on all hyper parameters and obtained 1,500 posterior samples from three parallel Monte Carlo Markov Chains of 20,000 iterations initiated at over-dispersed starting values. The first 5,000 iterations were discarded as burn-in and chains were thinned every 30th iteration to reduce auto-correlation and output size. Visual inspection of trace plots, and $\hat{R}$ of less than 1.1 confirmed convergence.

## Results

**Fig 2:**
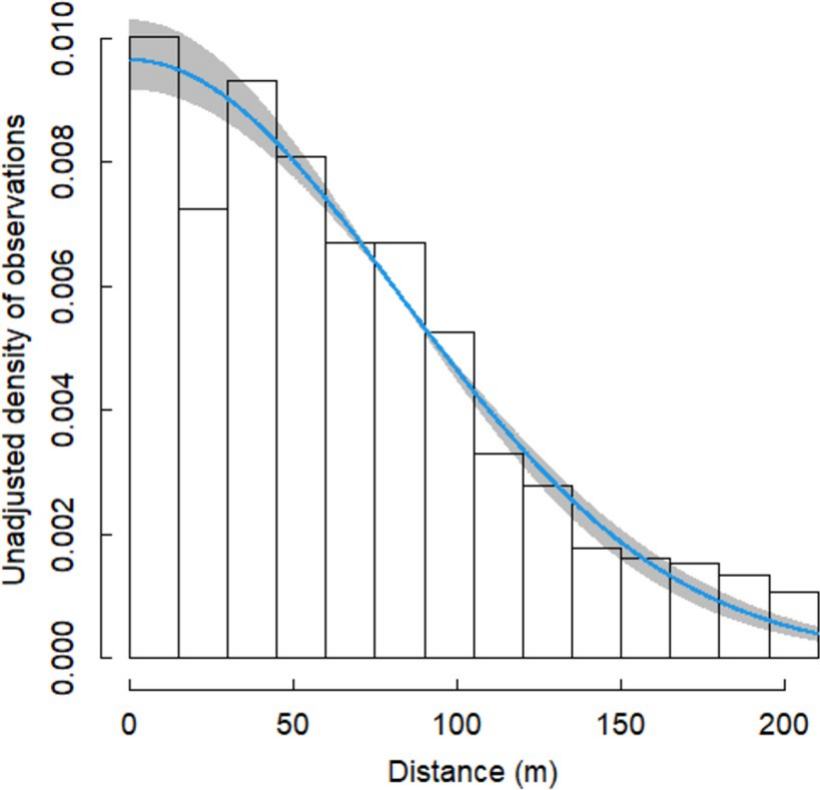
Murrelet detection function. Unadjusted density of marbled murrelet groups with distance from the transect centerline across all 2-month survey windows and years.

During the non-breeding season, we surveyed 32 PSUs in all 2-month survey windows/years except only 30 units were sampled in Nov-Dec of 2012, and 28 in Jan-Feb of 2012. Across all years, this resulted in our surveying a total of 789 PSUs and detecting 3,339 groups of murrelets. Overall, detection was 49.4% (46.3–52%) or expressed as sigma of the half normal detection curve (over a 210 m half width) it was 82.8% (95% CrI = 77.5–87.1%). The detection functions by year and survey window indicate little variation in the detection function across seasons or years (Fig 2).

For our year-round model, we used the 11 PSUs that were sampled in both the non-breeding and breeding seasons. For this analysis, we surveyed the 11 PSUs for all 37 year-survey window combinations, except that we only surveyed 10 and 9 units respectively in Nov-Dec and Jan-Feb of 2012. This resulted in a total of 404 sampling units surveyed and 2,133 groups of murrelets detected. Overall, detection was 46.0% (95% CrI = 42.8–49.1%) or expressed as sigma of the half normal detection curve (over a 210 m half width) it was 77.1% (95% CrI = 71.8–82.2%). Again, there was little variation in detection functions among years resulting in relationships almost identical to Fig 2 and, as a result, the functions are not presented.

**Fig 3:**
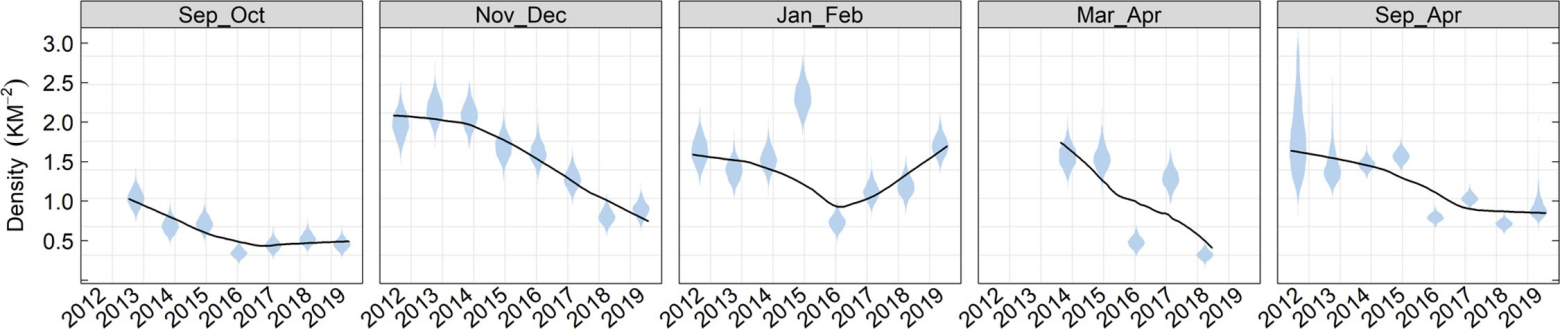
Marbled murrelet group size. Posterior median (points), 25–75% quartile (thick bars) and 95% credible interval (thin bars) of marbled murrelet group size by 2-month survey window for all years pooled. The dark boxplots with circles and the light plots with diamonds were derived from the non-breeding (Sep-Apr) model and year-round (Sep-Jul) model, respectively. The year-round model only includes the sampling units consistent between breeding and non-breeding seasons (n = 11) and the non-breeding model includes all 32 sampling units. For the most part, murrelets were detected as pairs or singles. As a result, we did not attempt to model the effect of group size on detection by distance.

Comparing average murrelet group size across two-month survey windows (all years pooled; Fig 3) suggests a gradual increase in group size between September and mid-Winter and then a decline with the lowest densities observed late in the breeding season (Jun-Jul). However, most group detections were of pairs for all two-month survey windows. Because of these seasonal differences in group size, we used season-specific group sizes in our density estimates.

**Fig 4:**
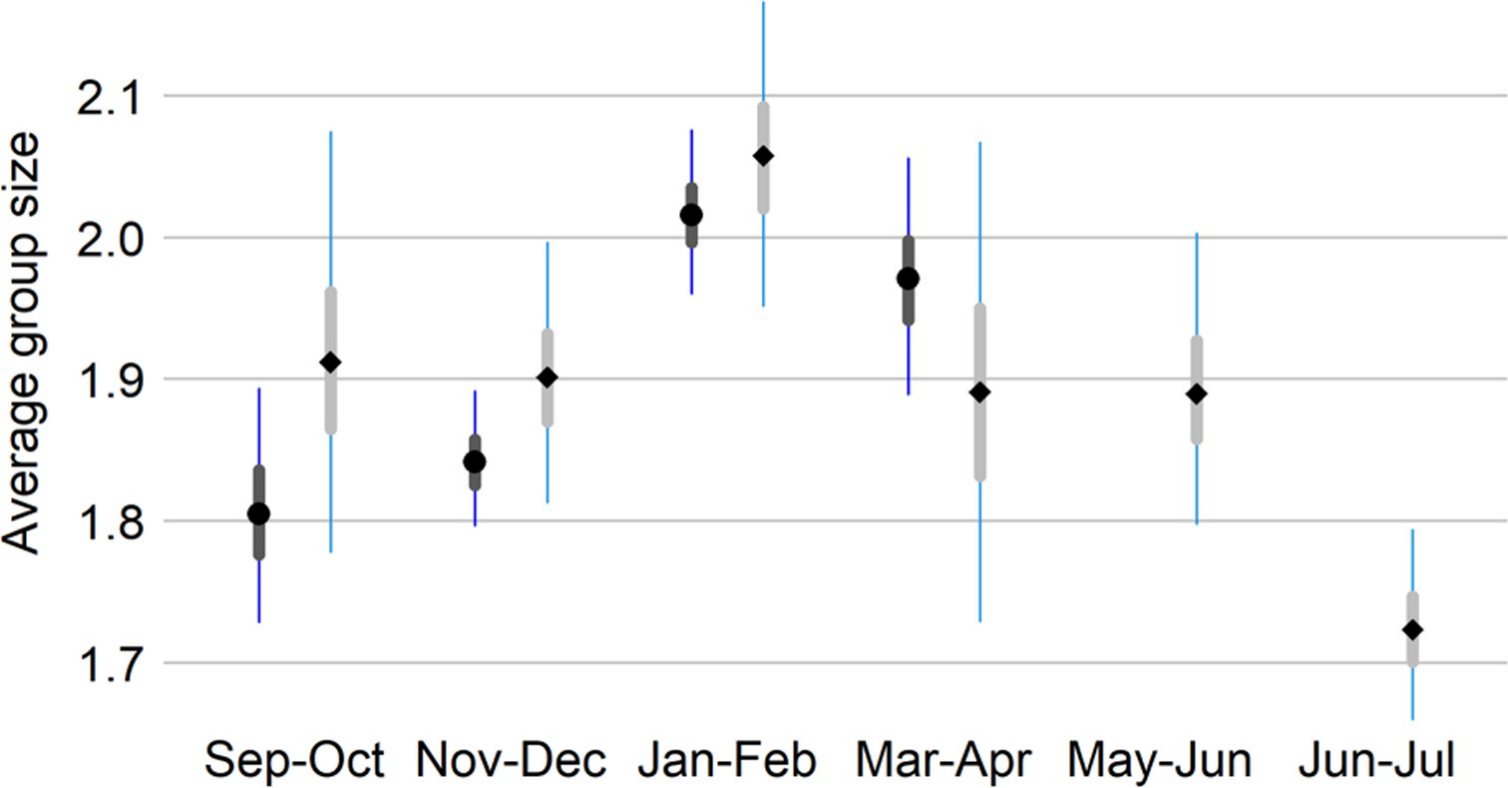
Marbled murrelet density by strata within survey season. Estimated (± 95% Crl) marbled murrelet density for each year and 2-month survey window combination in Strata 2,3,4, and 5.

For the non-breeding season analysis, Stratum 2 (Northwest study region, Admiralty Inlet) had the highest density in all seasons and years and Stratum 5 (Central Puget Sound) had few if any birds detected in any season/year combination (Fig 4). For example, the density of strata 3 and 4 in Sep and Oct was approximately 35–40% of that in stratum 2 and there were almost no murrelets detected in stratum 5. Between Jan and early-Apr nearly all murrelets are detected in Stratum 2. In general, densities are higher from late-Sep to Dec, low in all strata/year combinations in mid-winter (Jan-Feb) and they remain low for all strata/year combinations, except for high densities in Stratum 2 for three years (Fig 4).

**Fig 5:**
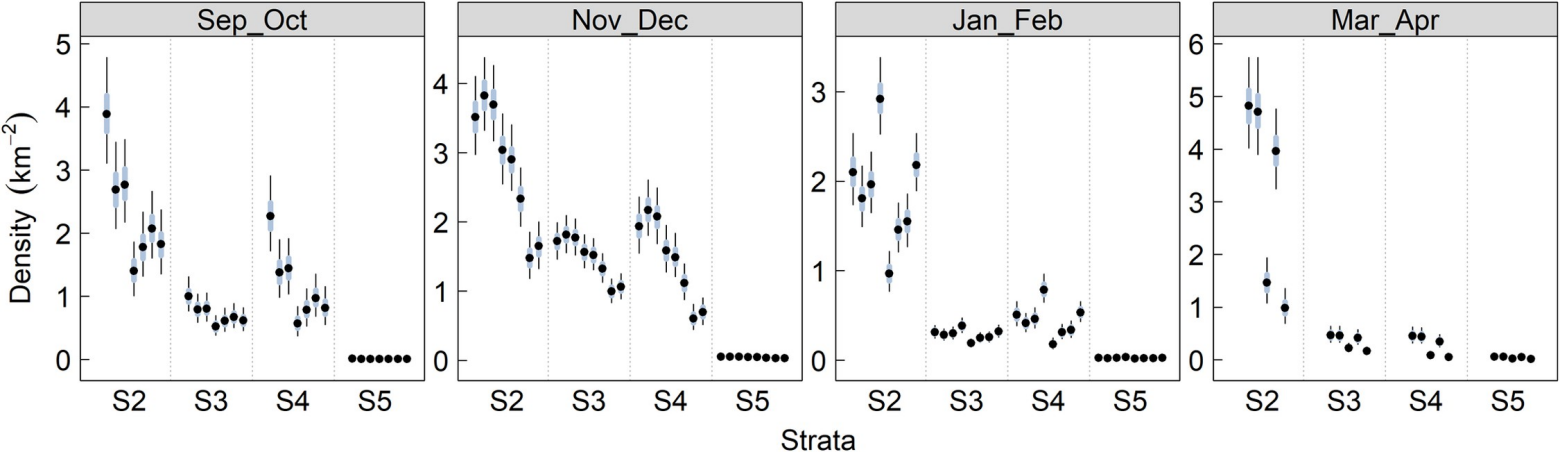
Relative marbled murrelet density for two 2-month breeding season survey intervals relative to that observed in the non-breeding season. A 100% relative density in May-Jun for a given year indicates that the density of murrelets was identical to that observed during the non-breeding season (Sep-Apr) and would suggest that birds are not moving into the region from outer coastal environments to molt and over-winter as we predicted. However, if the May-Jun density were 50% of that observed during the non-breeding season, then there would be evidence for movement into the region during the non-breeding season.

For each year, and when both breeding and non-breeding season data were available (2014, 2015, 2017), we compared the mean density from the two (May-Jun and Jun-Jul) breeding season survey windows to the mean density of the previous four 2-month non-breeding survey windows. In general, the mean density in May-Jun was similar to the non-breeding density except in 2014, when the breeding season density was about twice that of the non-breeding season (Fig 5). For the Jun-Jul survey window, the breeding season density was about half that of the winter in 2012, similar to the non-breeding density in 2013 and 2014, and nearly three times higher than the non-breeding density in 2015 and 2017 (Fig 5).

**Fig 6:**
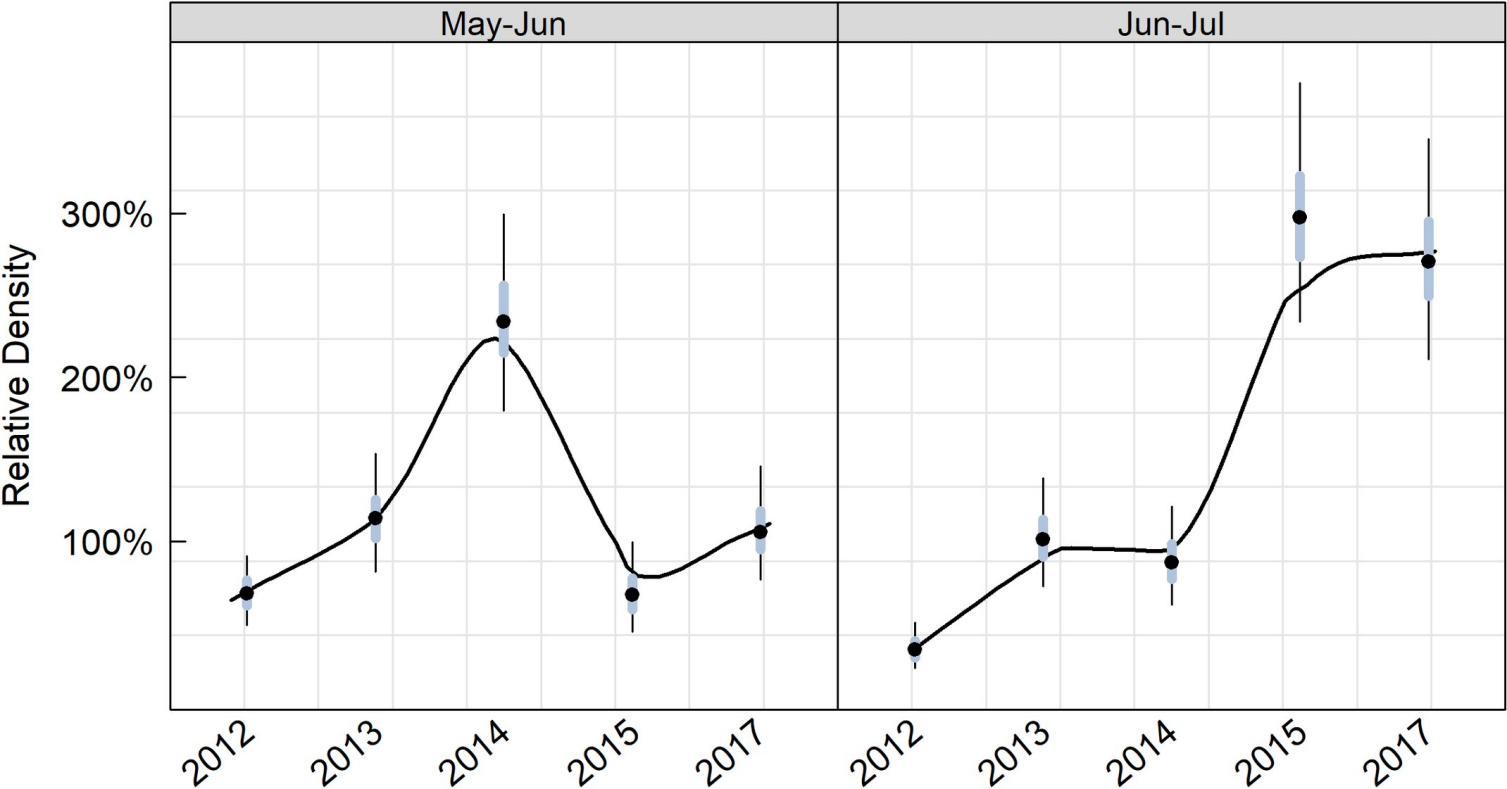
Marbled murrelet density by sampling window and year. Violin plot depicting the posterior distribution of annual marbled murrelet density (km^2^) by 2-month survey window during the non-breeding season. Black trend line derived from locally weighted sum of square regression (loess) fit to all posterior draws.

Looking at change in non-breeding murrelet density across all 8 years and strata combined, there is strong evidence of a decline across the entire non-breeding period (Sep-Apr) and in all seasons except for the Jan-Feb window where there was no apparent trend (Fig 6). It is also clear that there is a seasonal interaction, and that trend is non-linear within season (Fig 6). Given that the trend is non-linear, we thought it most appropriate to compare the change between the first year (2012–2013) and last year (2019–2020) for each 2-month survey windows. Using this approach, murrelet density declined by 55–56% in the fall survey windows (Sep-Oct, Nov-Dec), there was no change in mid-winter density (Jan-Feb) (Table 1) and a 79.5% decline in spring density (Mar-Apr).

**Table 1:** Percent change (95% Crl) in predicted murrelet density between the first and last year surveyed for each 2-month survey window and for the entire (Sep-Apr) non-breeding season.

Sep-Oct	Nov-Dec	Jan-Feb	Mar-Apr	Sep-Apr
-56.0% (-69.0 –-38.9)	-54.6% (-65.5– -41.8)	3.9% (-18.4–33.3)	-79.5% (-85.8 –-70.5)	-48% (-69.6 –-13.6)

## Discussion

Across the 8 years, we observed strong non-breeding declines in murrelet density. These declines were most pronounced in the fall (Sep-Oct, Nov-Dec) and late winter/spring (Mar-Apr), and with essentially no change in murrelet density in mid-winter (Jan-Feb) (Table 1). To put our results in context, we summarize other survey efforts that have examined murrelet trends in the Salish Sea, and adjacent habitats on the Washington Coast and coastal British Columbia (Table 2). All regional breeding season survey efforts (from radar to boat-based) also indicate long-term population declines. The only regional non-breeding surveys that we are aware of are land-based point counts conducted by citizen scientists using standardized protocols in both Washington State and in coastal British Columbia (Table 2). The Canadian effort yield mixed results with increases in murrelet detections in the Strait of Georgia and Strait of Juan de Fuca and declines along the outer B.C. coast [27]. The credible intervals for both trends overlap zero, indicating weak evidence for positive or negative trends. The US effort indicates an increase in probability of occurrence (2007–2013; [28]). Because this analysis focused on occurrence rather than abundance, the authors cautioned that declining species may exhibit a less aggregated spatial distribution, resulting in their probability of detection increasing without an increase in abundance [28]. Murrelets were rarely detected by this effort (probability of occurrence very close to zero). Results may also differ from our study because there was very little space or time overlap with our work. Interestingly, the north-south gradient in murrelet abundance that we observed was also apparent in Ward et al.’s [28] occupancy study, with higher murrelet site occupancy in north Puget Sound when compared to south Puget Sound.

**Table 2:** Pacific northwest marbled murrelet trend by region, season, time-period and survey method.

Study	Region	Time Period	Season	Methods	Results
**McIver et al. 2021 [4]**	U.S. Salish Sea, USA	2001–2020	Breeding	Boat-based, line transect	-5.0%/yr (95%CI: -7.0 –-2.9)
	Washington coast, USA	2001–2019			-2.2%/yr (95%CI: -5.7–1.5)
**Lorenz and Raphael 2018 [29]**	San Juan Archipelago, WA, USA	1995–2012	Breeding	Boat-based, line transect	-3.9%/yr (95% CI: -5.7 –-0.4).
**Bertram et al. 2015 [30]**	Coast-wide, B.C., Canada	1996–2013	Breeding	Radar detections	-1.6%/yr (95% Crl -3.2 –- 0.01)
	E. Vancouver Island, B.C., Canada				-8.6%/yr (95% Crl: -1.3–11.0)
	S. Mainland Coast, B.C., Canada				-3.1%/yr (95% Crl: -5.8 –-0.5)
**Drever et al. 2021 [31]**	Coast-wide, B.C., Canada	1996–2018	Breeding	Radar detections	-2.4%/yr (95%CI: -3.3 –-1.4)
	E. Vancouver Island, B.C., Canada				-7.0%/yr (95%CI: -10.7 –-3.4)
	S. Mainland Coast, B.C., Canada				-3.8%/yr (95%CI: -5.4 –-2.2)
**Ethier et al. 2020 [27]**	Strait of Georgia and Strait of Juan de Fuca, B.C., Canada	1999–2019	Non-breeding	Shore-based counts from citizen scientists, standardized protocols	+3.36%/yr (95%Crl: −1.29–8.00)
	Outer coast, B.C., Canda				-7.5%/yr (95%Crl: −16.72–2.74)
**Ward et al. 2015 [28]**	Puget Sound	2007–2013	Non-breeding	Shore-based counts from citizen scientists, standardized protocols	Increase in probability of occurrence

Both our strata and murrelet density are roughly arranged north to south with the most northerly stratum (2) having the highest murrelet density, the most southerly stratum (5) having the lowest density, The murrelet density gradient mirrors the north-south gradient in the biomass of important forage fish species for murrelets (Pacific herring, surf smelt [*Hypomesus pretiosus*], and Pacific sand lance [*Ammodytes personatus*]) in Puget Sound, which have higher densities in the north [32, 33]. In Central Puget Sound, where we detected few murrelets, the abundance of Pacific herring and surf smelt have declined by up to two orders of magnitude between the early 1970s and 2011 [33]. We are not suggesting a cause-and-effect relationship between changes in murrelet and prey abundance, but we are recommending that this potential relationship is a hypothesis worthy of a quantitative investigation.

Beyond the potential negative effects on murrelet prey, there is some evidence that the collective activities of humans may also negatively influence marbled murrelet abundance and distribution. Raphael et al. [7, 21] evaluated the relative influence of marine and terrestrial factors on the distribution and abundance of murrelets at-sea during the breeding season. They found that changes in the amount of higher suitability nesting habitat was the best predictor of changes in murrelet abundance and distribution in the Salish Sea during the breeding season. However, unlike other surveyed areas in Oregon and northern California, the next best predictor of changes in murrelet abundance and distribution was the marine human footprint, which reflects more intense vessel traffic, fishing pressure, and pollution. For the closely related Kittlitz’s murrelet (*Brachyramphus brevirostris*), vessel activity alone can result in a 30-fold increase in flight behavior, which is energetically costly [34]. The relative importance of these human factors on non-breeding season marbled murrelet distribution and abundance remains to be investigated.

When examining fall through winter patterns of abundance, it is critical to consider the influence of marbled murrelet natural history. Like most alcids, marbled murrelets undergo a nearly simultaneous prebasic flight-feather molt that renders them flightless for several weeks following the breeding season [9]. In Barkley Sound, British Columbia, most individuals disperse before the prebasic molt [9] and, for some populations, post-breeding murrelets may disperse hundreds of kilometers after breeding [12, 35]. In other populations, murrelets appear to use protected and highly productive upwelling areas near breeding sites for the prebasic molt and then disperse hundreds of kilometers after molting [11]. These variations in post-breeding dispersal pattern occur even though the timing of molt appears to be similar among regions (central California: early Aug through Nov; Barkley Sound: mid-Jul to mid-Nov) [9]. We predicted that murrelets might move into the relatively protected waters of Puget Sound to molt and over-winter (e.g., [35]). However, our results indicate relatively low Puget Sound murrelet densities during the period when molting occurs (Sep-Oct) and strong declines during the molting window across years, suggesting that birds may be moving away from Puget Sound due to decreased habitat quality locally, or improved habitat quality elsewhere [36].

## Conclusion

Across the 8 years of non-breeding season survey effort, we found strong evidence for declines in Puget Sound marbled murrelets in the fall to early winter (Sep–Dec), suggesting a decline in the use of this habitat when post-breeding dispersal and molt commonly occurs, and declines in the spring (Mar-Apr). This decline, coupled with the decades-long breeding-season declines for this same region [4], suggests either that the population is declining or that birds are moving out of the region due to a decline in habitat quality locally or improved conditions elsewhere, or both (e.g., [36]). We observed 55–56% declines in the fall and early winter (Sep-Dec) during 8 years of surveys and the breeding season density of murrelets in this region has declined by over half (by over 80% in the high-density areas) between 2002 and 2018 [4]. We found no evidence that birds are moving into the protected waters of Puget Sound to molt or over-winter as predicted. Instead, the relative density during the breeding season is generally similar to or higher than what we observed in the fall and winter. We encourage future investigations to examine the various factors that might influence post-breeding dispersal including increased energetic demands of molt, changes in the abundance and distribution of small prey, and the need for protected areas away from potential predators during the flightless molt period.

There is evidence of long-term declines in the biomass of important forage fish (e.g., smelt and Pacific herring) in the southern portion of Puget Sound [33] where we observed very few or no murrelets. However, the loss of important forage fish in south Puget Sound is complicated by changes in forage fish in north Puget Sound and an apparent increase in anchovy during recent warm water events. To better understand these complex interactions, we recommend research focused on evaluating the relationship between water quality, forage fish spawning habitat and biomass, physical drivers such as sea surface temperature, and murrelet abundance and trends. To accomplish this, a year-round forage fish survey effort is needed. At the same time, the importance of Admiralty Inlet (Stratum 2) relative to other areas surveyed in this study, suggest that there are “hotspots” of abundance in the waters around Marrowstone Island south to Port Ludlow (see detection rate by PSU in S1 Table) that may be important to conservation and long-term management of the species in the Puget Sound region. These hotspots may be facilitated by bathymetric features that enhance foraging opportunities in areas with greater forage fish abundance.

## Supporting information

S1 Table Average annual detection rate (murrelets/km^2^). Sample size (n) indicates number of years surveyed.
(XLSX)
