# Correction: Non-breeding changes in at-sea distribution and abundance of the threatened marbled murrelet (*Brachyramphus marmoratus*) in a portion of its range exhibiting long-term breeding season declines

**DOI:** 10.1371/journal.pone.0310567

**Published:** 2024-09-12

**Authors:** Scott F. Pearson, Ilai Keren, Monique M. Lance, Martin G. Raphael

The images for Figs 3 to 6 are incorrectly switched. The image that appears as Fig 3 should be Fig 6, the image that appears as Fig 4 should be Fig 3, the image that appears as Fig 5 should be Fig 4 and the image that appears as Fig 6 should be Fig 5. The figure captions appear in the correct order.

**Fig 3:**
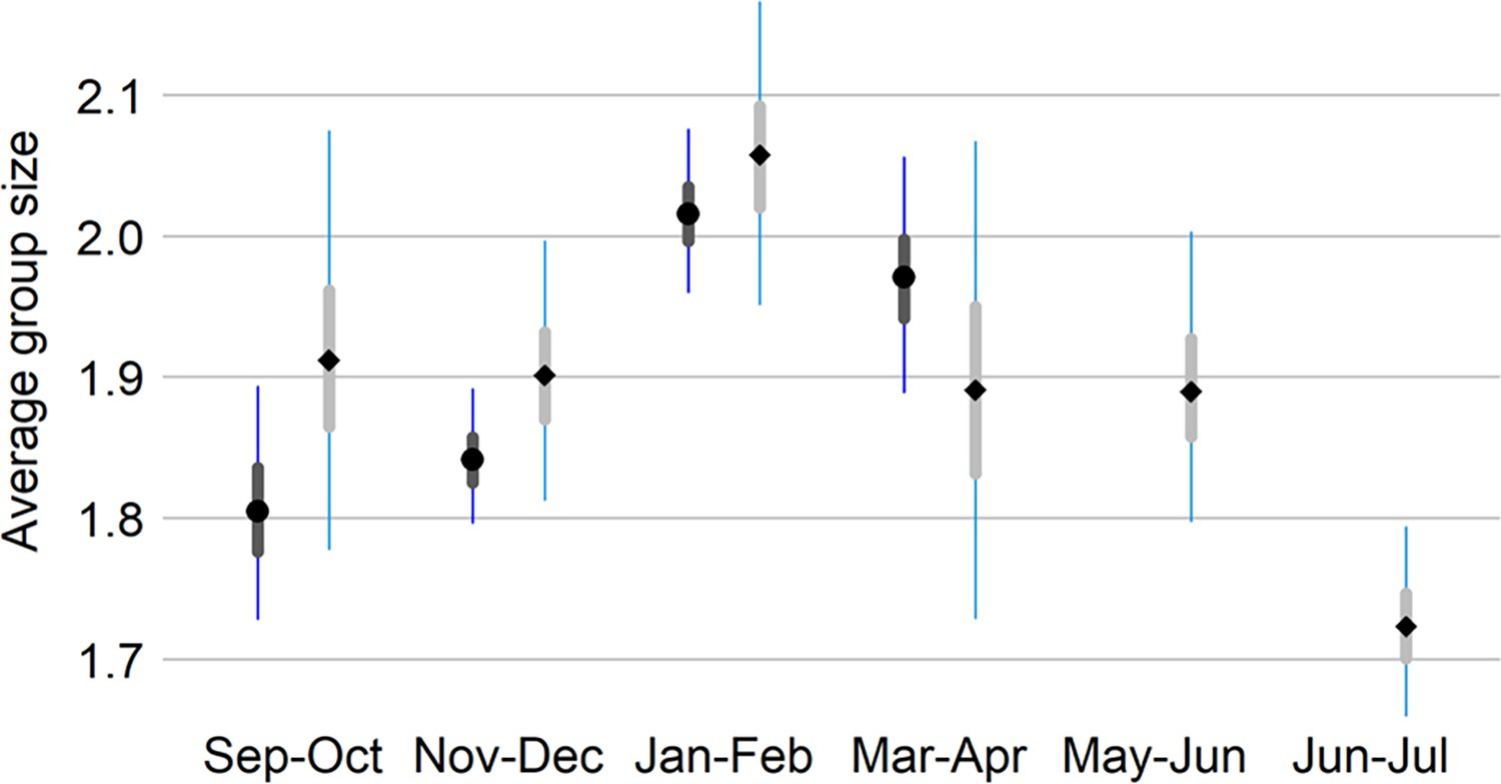
Marbled murrelet group size.

Posterior median (points), 25–75% quartile (thick bars) and 95% credible interval (thin bars) of marbled murrelet group size by 2-month survey window for all years pooled. The dark boxplots with circles and the light plots with diamonds were derived from the non-breeding (Sep-Apr) model and year-round (Sep-Jul) model, respectively. The year-round model only includes the sampling units consistent between breeding and non-breeding seasons (n = 11) and the non-breeding model includes all 32 sampling units. For the most part, murrelets were detected as pairs or singles. As a result, we did not attempt to model the effect of group size on detection by distance.

**Fig 4:**
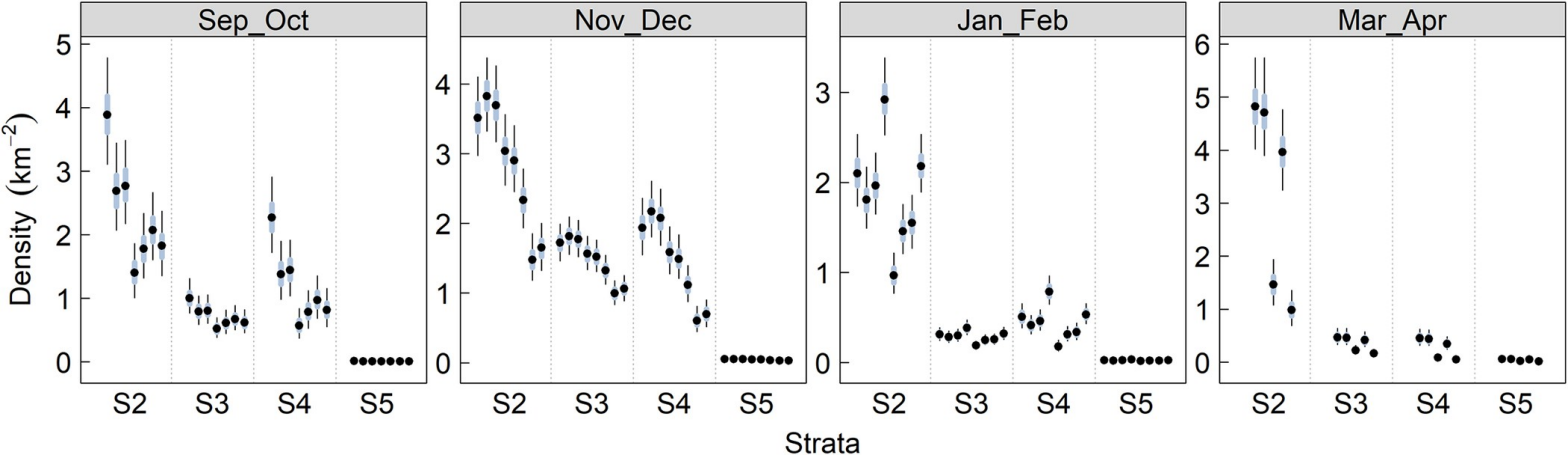
Marbled murrelet density by strata within survey season.

Estimated (± 95% Crl) marbled murrelet density for each year and 2-month survey window combination in Strata 2,3,4, and 5.

**Fig 5:**
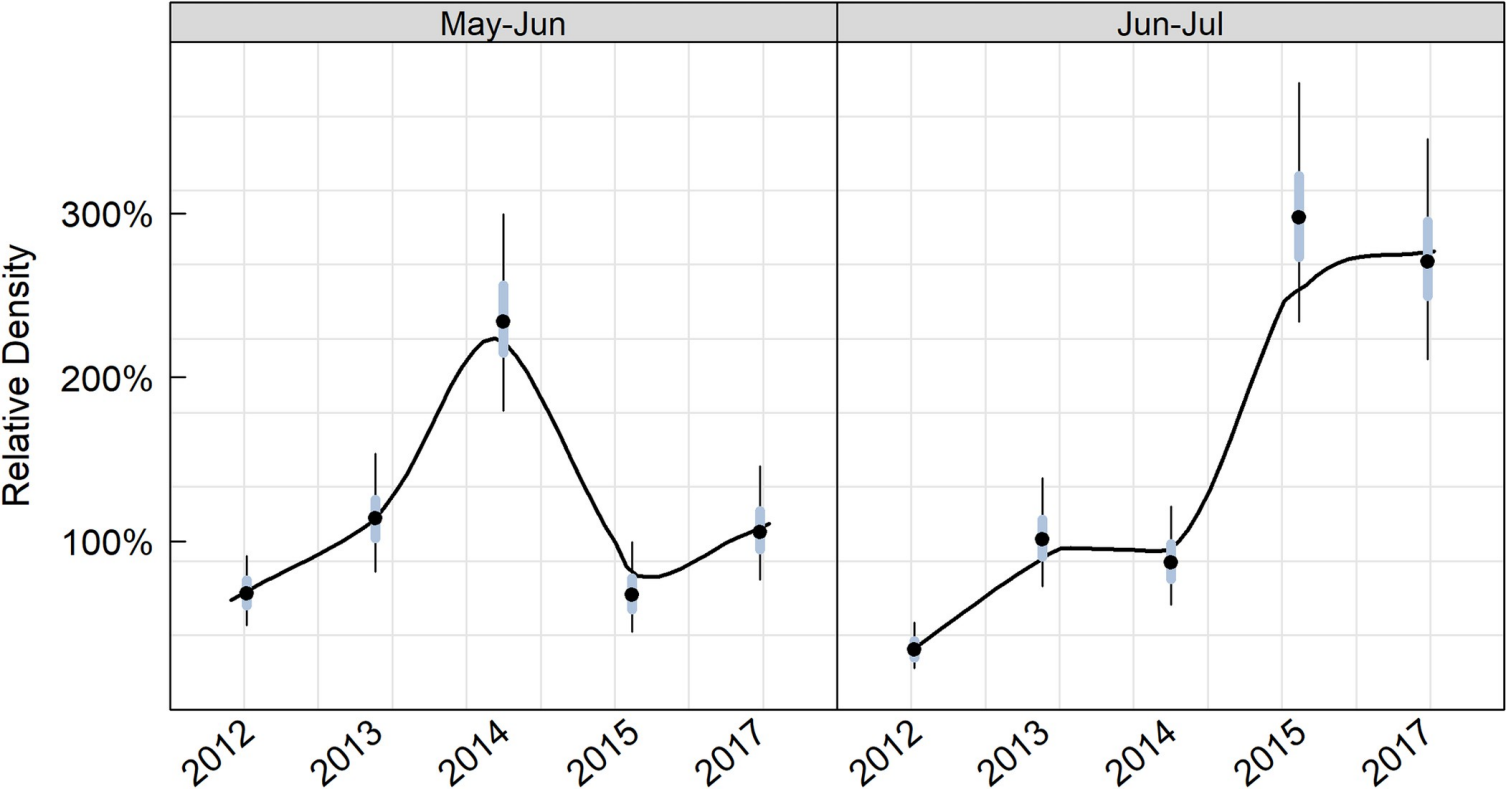
Relative marbled murrelet density for two 2-month breeding season survey intervals relative to that observed in the non-breeding season.

A 100% relative density in May-Jun for a given year indicates that the density of murrelets was identical to that observed during the non-breeding season (Sep-Apr) and would suggest that birds are not moving into the region from outer coastal environments to molt and over-winter as we predicted. However, if the May-Jun density were 50% of that observed during the non-breeding season, then there would be evidence for movement into the region during the non-breeding season.

**Fig 6:**
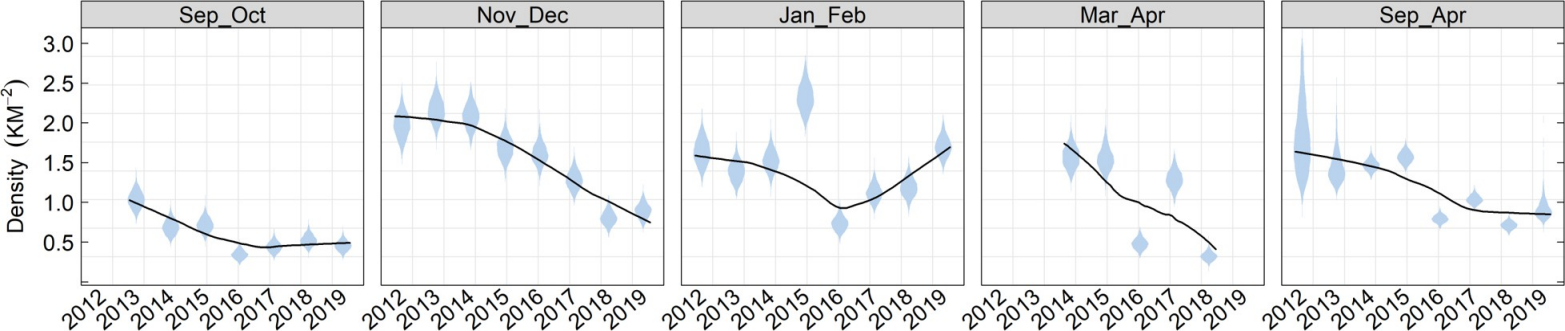
Marbled murrelet density by sampling window and year.

Violin plot depicting the posterior distribution of annual marbled murrelet density (km^2^) by 2-month survey window during the non-breeding season. Black trend line derived from locally weighted sum of square regression (loess) fit to all posterior draws.
